# Transcatheter edge-to-edge repair for severe mitral regurgitation in a patient with a massive left atrium: a case report

**DOI:** 10.1093/ehjcr/ytag222

**Published:** 2026-03-20

**Authors:** Qingwei Ni, Zhijie Mao, Changxi Chen, Xingxing Chen, Hao Zhou

**Affiliations:** Department of Cardiovascular Medicine, The First Affiliated Hospital of Wenzhou Medical University, Nanbaixiang Street, Ouhai District, Wenzhou, Zhejiang 325000, China; Department of Cardiovascular Medicine, The First Affiliated Hospital of Wenzhou Medical University, Nanbaixiang Street, Ouhai District, Wenzhou, Zhejiang 325000, China; Department of Cardiovascular Medicine, The First Affiliated Hospital of Wenzhou Medical University, Nanbaixiang Street, Ouhai District, Wenzhou, Zhejiang 325000, China; Department of Cardiovascular Medicine, The First Affiliated Hospital of Wenzhou Medical University, Nanbaixiang Street, Ouhai District, Wenzhou, Zhejiang 325000, China; Department of Cardiovascular Medicine, The First Affiliated Hospital of Wenzhou Medical University, Nanbaixiang Street, Ouhai District, Wenzhou, Zhejiang 325000, China

**Keywords:** Mitral regurgitation, Transcatheter edge-to-edge repair, Giant left atrium, MitraClip, Case report

## Abstract

**Background:**

Transcatheter edge-to-edge repair (TEER) poses significant technical challenges in patients with a massive left atrium (LA), where conventional anatomical criteria often deem the procedure unsuitable.

**Case summary:**

A 77-year-old man, at prohibitive surgical risk, presented with a giant LA (113 × 129 × 133 mm) and severe mitral regurgitation (MR). After pacemaker implantation, TEER was performed. Despite suboptimal echocardiographic windows and challenging leaflet capture, a tailored posteroinferior transseptal puncture 4.67 cm above the mitral annular plane provided a stable trajectory, enabling successful navigation and deployment of three MitraClip devices (Abbott, Santa Clara, CA, USA). The procedure achieved an excellent outcome with a mean gradient of 5 mmHg and only mild residual MR. Marked left atrial reverse remodelling and symptomatic improvement were observed at 1-month follow-up.

**Discussion:**

This case demonstrates that TEER is a viable and effective intervention for patients with extreme LA enlargement, challenging conventional anatomical selection criteria. Success hinges on technical precision—particularly an optimized transseptal puncture—and prioritizing favourable haemodynamic outcomes over rigid anatomic thresholds.

Learning points

- Massive left atrial enlargement should not be considered an absolute contraindication to transcatheter edge-to-edge repair (TEER).
- An optimized, low posterior transseptal puncture height is crucial for device navigation in giant left atria.

## Introduction

Severe mitral regurgitation (MR) remains a major contributor to morbidity and mortality, particularly in elderly patients with significant comorbidities.^1^ While transcatheter edge-to-edge repair (TEER) offers a less invasive alternative with favourable outcomes for high-risk surgical candidates,^2^ its application in patients with a massively enlarged left atrium (LA) is associated with distinct technical challenges.^3^ Cases involving mixed-aetiology MR, particularly the combination of degenerative pathology and atrial functional MR as seen in our patient, pose additional complexity. This report describes the successful management of such a high-risk case, highlighting the technical adaptations and decision-making process.

## Summary figure

**Figure:**
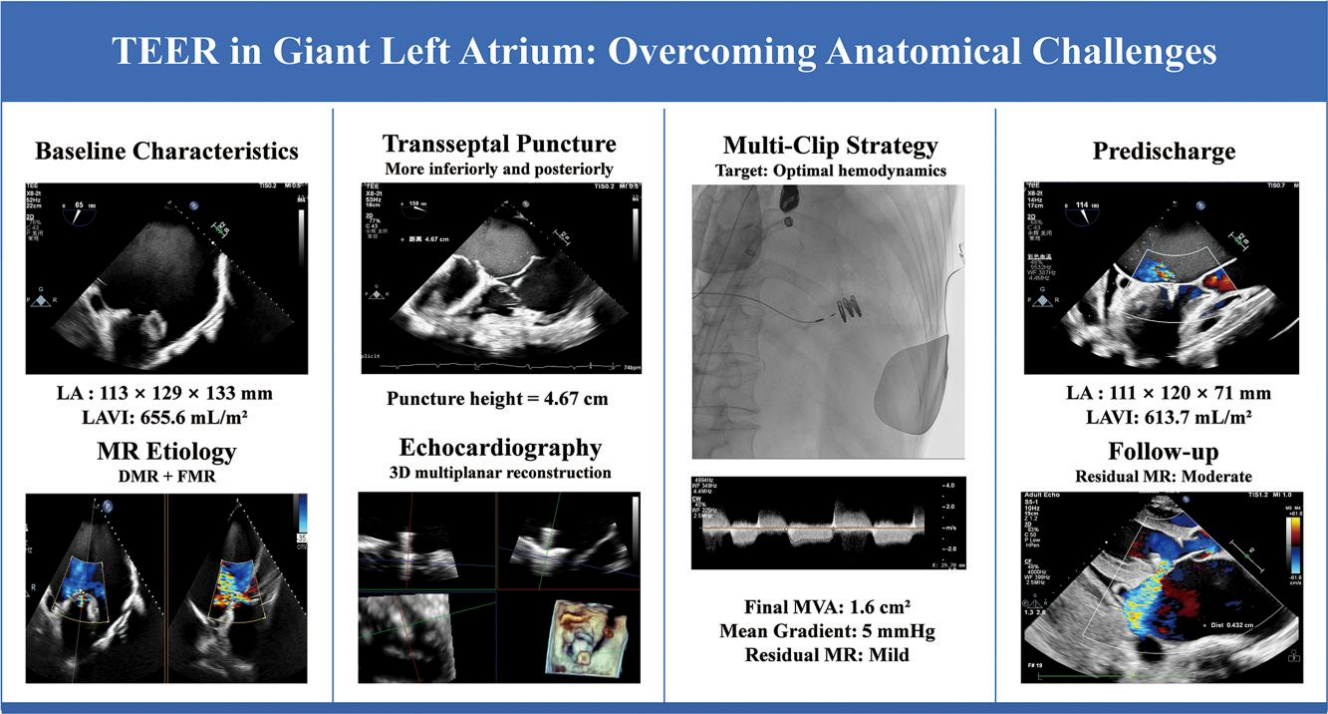
Schematic illustration of the patient’s baseline presentation, key technical challenges, and post-procedural outcomes following transcatheter edge-to-edge repair (TEER).

## Case presentation

**Figure 1:**
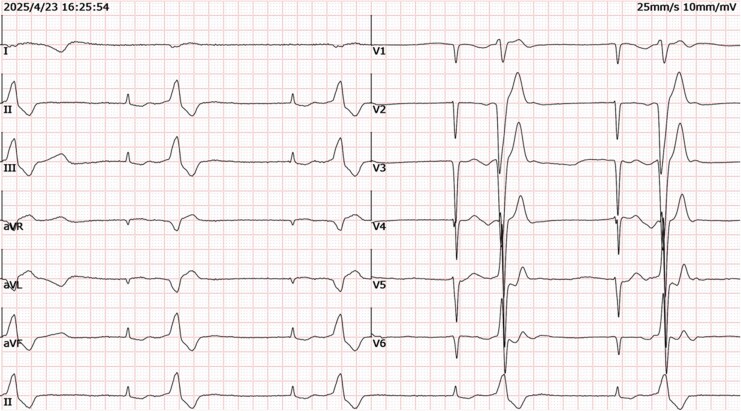
Baseline electrocardiogram. Tracing showing atrial fibrillation with high-grade atrioventricular block, a junctional escape rhythm, and ventricular bigeminy.

A 77-year-old man with hypertension, atrial fibrillation, and chronic heart failure presented with chest tightness and fatigue for 1 month. Admission blood pressure was 101/64 mmHg. Electrocardiography showed atrial fibrillation with high-grade atrioventricular (AV) block (*Figure 1*), attributed to digoxin toxicity (level 4.4 ng/mL; therapeutic range 0.5–2.0 ng/mL, falling to 0.8 ng/mL after withdrawal). Serum potassium was 3.81 mmol/L (normal range 3.5–5.0 mmol/L). Persistent high-grade AV block required permanent pacemaker implantation.

**Figure 2:**
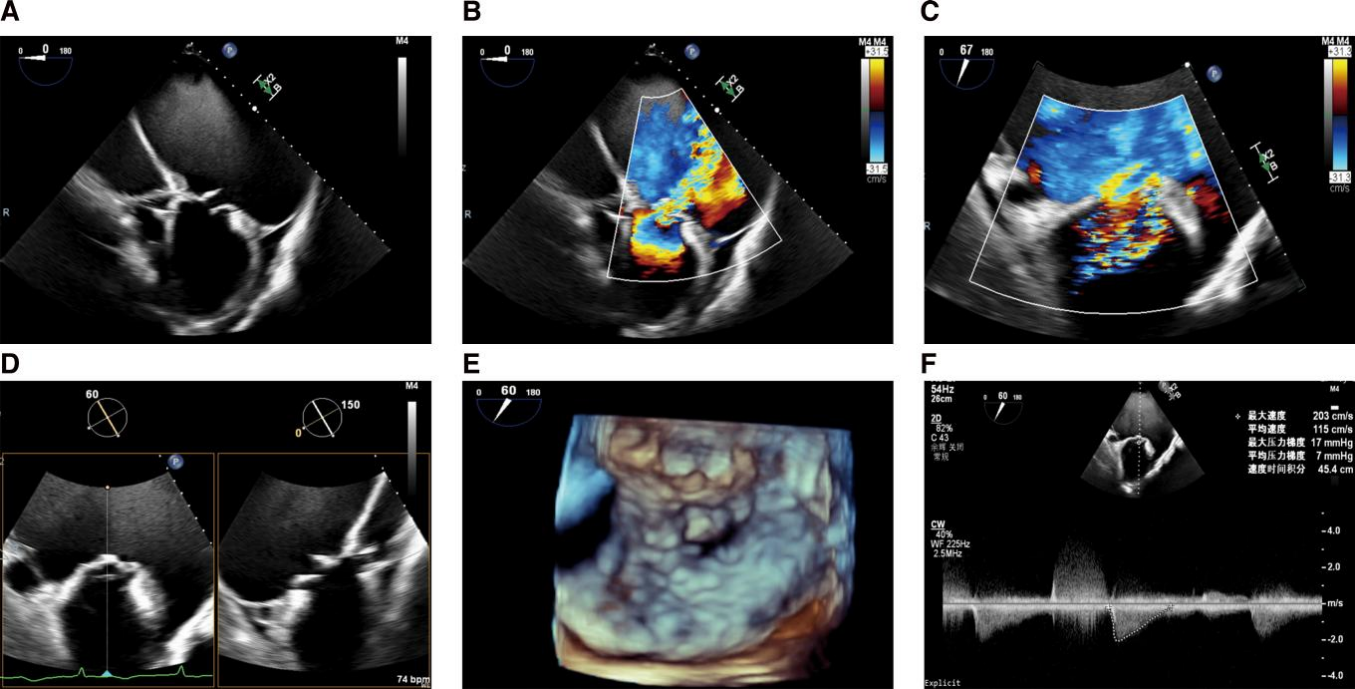
Preprocedural transoesophageal echocardiography. (*A–F*) Preprocedural assessment demonstrating severe mitral regurgitation due to anterior and posterior leaflet prolapse at the A2–P2 segment.

Echocardiography revealed preserved left ventricular systolic function, with a left ventricular ejection fraction (LVEF) of 66.9%, and significant chamber enlargement, including massive dilation of the left atrium (113 × 129 × 133 mm)—resulting in a left atrial volume index (LAVI) of 655.6 mL/m^2^. Severe MR of mixed aetiology was identified, involving anterior and posterior leaflet prolapse. The primary mechanism was a flail of the A2 scallop (flail width 24 mm, flail height 12 mm), which generated a wide, eccentric, and posteriorly directed regurgitant jet, compounded by severe annular dilation contributing to an atrial functional component. Transoesophageal echocardiography (TEE) using three-dimensional (3D) multiplanar reconstruction confirmed a mitral valve orifice area of 4.12 cm^2^ with a mean gradient of 7 mmHg at 86 bpm (*Figure 2*, Supplementary material online, *Video S1*), severe annular dilation (anterior–posterior diameter of 44 mm and an anterolateral–posteromedial diameter of 39 mm), an effective regurgitant orifice area of 2.85 cm^2^, and systolic pulmonary venous flow reversal. Coronary angiography excluded obstructive disease. Laboratory studies noted elevated NT-proBNP (1019 ng/L; normal <125 ng/L) and thrombocytopenia (69 × 10^9^/L; normal 150–450 × 10^9^/L). The heart team assessed a very high surgical risk (Society of Thoracic Surgeons score 17.43%), attributing his decline to severe MR and AV block. A permanent pacemaker was implanted, followed by planned TEER.

**Figure 3:**
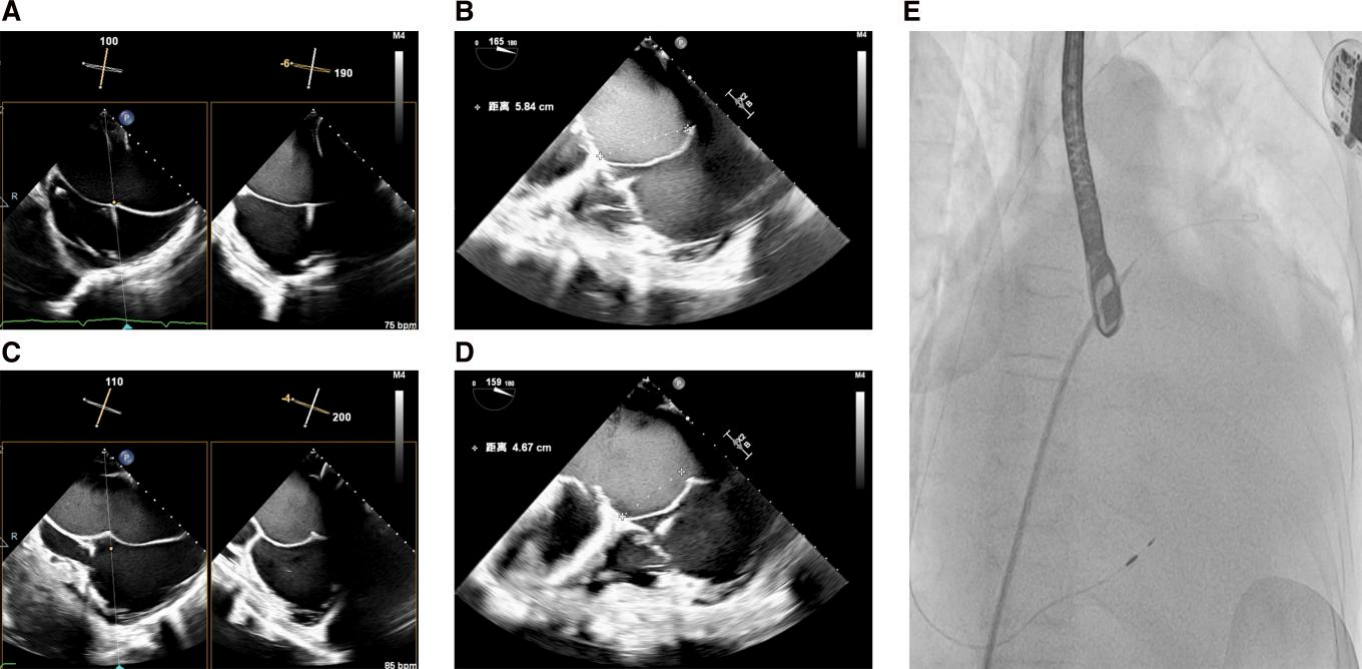
Transseptal puncture procedure. (*A*, *B*) Initial transseptal puncture at a suboptimal height of 5.84 cm. (*C*, *D*) Final puncture site at an optimized height of 4.67 cm following needle repositioning toward the inferior vena cava (IVC) orifice. (*E*) Fluoroscopic visualization of the guidewire position.

TEER was performed under general anaesthesia. The massively enlarged left atrium caused suboptimal acoustic windows. An initial transseptal puncture was high (>5 cm); therefore, the needle was redirected inferiorly and posteriorly toward the inferior vena cava (IVC) orifice, achieving an optimal height of 4.67 cm (*Figure 3*).

**Figure 4:**
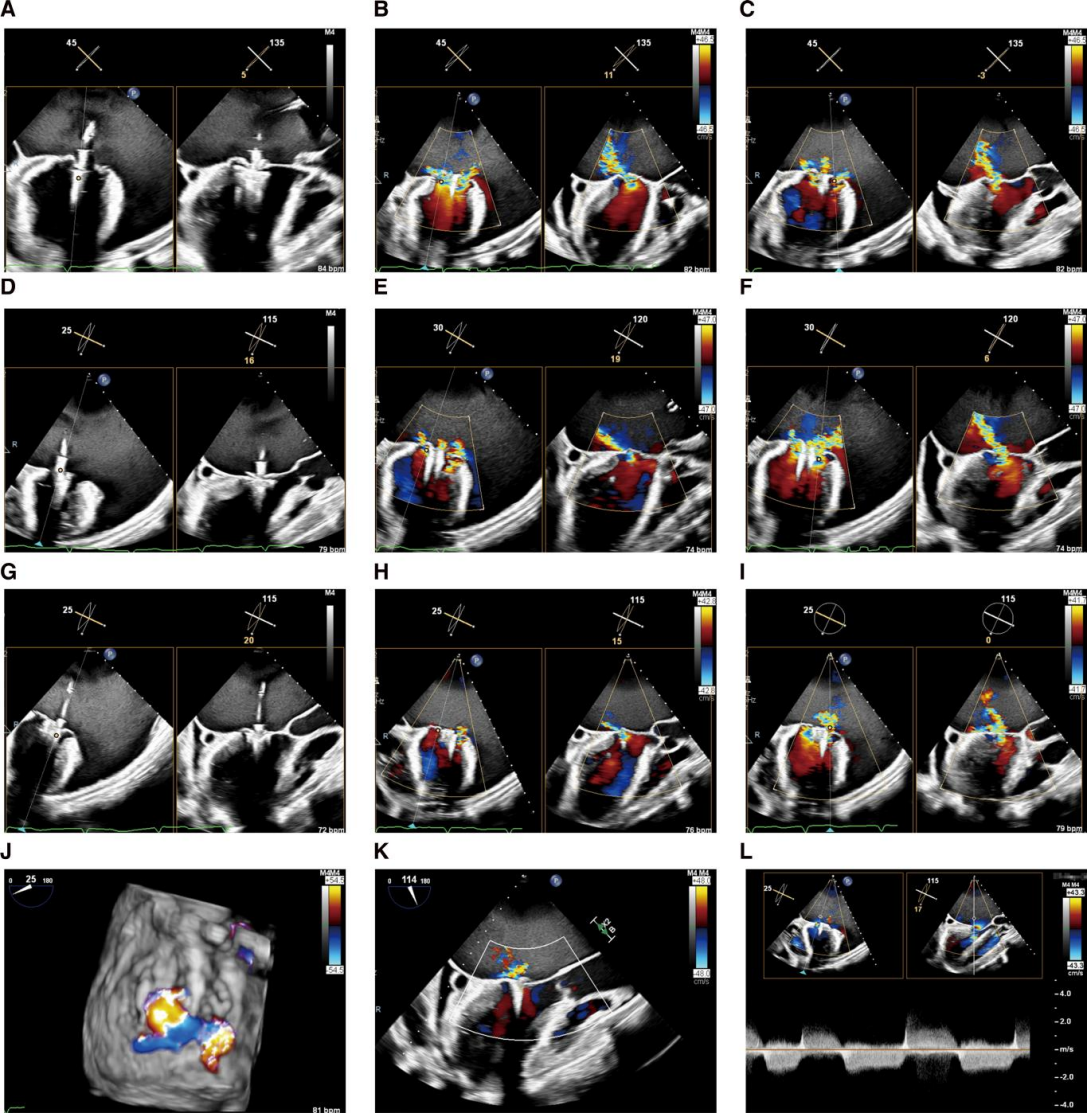
Intraprocedural transesophageal echocardiogram. (*A–C*) Residual medial and lateral regurgitation following deployment of the first MitraClip XTW device. (*D–F*) Reduction in regurgitation after implantation of a second MitraClip XT device medial to the first clip. (*G–I*) Further reduction after deployment of a third MitraClip XT device lateral to the first clip. (*J–L*) Final result showing mild residual mitral regurgitation and a mean transmitral gradient of 5 mmHg.

Profound atrial dilation required extensive manoeuvering to position the MitraClip XTW (Abbott, Santa Clara, CA, USA) above the mitral valve. Leaflet capture at the A2-P2 segment was challenging, requiring multiple attempts. After the first clip deployment, moderate residual regurgitation persisted medially and laterally. A second MitraClip XT was implanted medially, but a lateral jet near A1 remained. Despite concerns about valve area, a third XT clip was deployed laterally (*Figure 4*), resulting in a final mitral valve area of 1.6 cm^2^ with a mean gradient of 5 mmHg and a reduction in left atrial V-wave pressure (24 to 18 mmHg).

The patient was discharged on postoperative Day 7 without complications. Medications included rivaroxaban, dapagliflozin, torasemide, and spironolactone. Predischarge echocardiography showed preserved LV function, reduced left atrial size (111 × 120 × 71 mm; LAVI 613.7 mL/m^2^), stable clips, and mild residual MR. At 1 month, follow-up transoesophageal echocardiography revealed moderate (2+) residual MR. This integrative assessment was based on a vena contracta width of 4.3 mm and a regurgitant jet area to left atrial area ratio of 27% (33.8/126 cm^2^), and the mean transmitral gradient was 8 mmHg at a heart rate of 82 bpm (see Supplementary material online, *Video S2*).

## Discussion

This case successfully expands the conventional boundaries for TEER by demonstrating its feasibility in one of the largest reported left atria. Although consensus documents propose criteria for unsuitable anatomies (‘red-light’ category) based on experience with earlier devices,^4^ our experience suggests that LA size alone should not constitute an absolute contraindication. Careful heart team evaluation remains essential given the limited evidence on long-term outcomes. The observed left atrial reverse remodelling further supports TEER’s role in reducing atrial volume overload, though long-term benefits require further investigation.^5^

Performing TEER in a massively enlarged atrium introduces three principal technical challenges. First, the anatomic challenge of transseptal puncture is significant. Transseptal puncture requires a specifically tailored inferior and posterior approach to avoid complications and ensure adequate needle curvature.^6^ Excessively high punctures (>5 cm) risk preventing the clip delivery system from reaching the mitral valve plane.^7^ To circumvent this, we strategically redirected the needle near the IVC orifice, achieving an optimal height of 4.67 cm. Second, device manoeuvering and leaflet capture are challenged by the increased distance to the mitral valve, demanding extensive catheter manipulation. This difficulty, compounded by frequent annular dilation, makes achieving a stable and sufficient leaflet grasp particularly demanding. In such scenarios with poor coaptation, adjunctive techniques such as adenosine-induced transient cardiac arrest can be considered to temporarily stabilize the leaflets and facilitate grasping.^8^ Third, profound atrial dilation impairs ultrasound transmission, leading to suboptimal acoustic windows. In these situations, 3D-MPR can be instrumental in generating a clearer anatomical interface for guidance.^9^

Our clip strategy was tailored to the severe prolapse, characterized by a broad regurgitant jet (24.4 mm) and sufficient leaflet length (anterior 32 mm, posterior 21 mm) in the A2-P2 segment. Consequently, a MitraClip XTW was deployed as the first device. Despite multiple grasping attempts to optimize leaflet capture, residual medial and lateral jets persisted due to the extensive nature of the prolapse and annular dilation. This necessitated the sequential implantation of two additional MitraClip XT devices—first medially and then laterally to the initial clip—to address the residual regurgitation. This multi-clip strategy, which prioritized adequate regurgitation reduction over rigid adherence to a pre-defined valve area, resulted in a favourable haemodynamic outcome with only mild residual MR and a mean gradient of 5 mmHg. This outcome underscores that an integrated haemodynamic assessment should supersede reliance on isolated anatomic metrics.^10^

Managing concomitant conditions was also crucial in this case. While pacemaker implantation for AV block was routine, thrombocytopenia posed a profound dilemma for long-term anticoagulation in atrial fibrillation. A cautious regimen of rivaroxaban 10 mg daily was implemented, successfully balancing stroke prevention against bleeding risk as evidenced by stable early post-procedural haemoglobin. This dilemma highlights the potential role of future hybrid strategies combining TEER with left atrial appendage closure,^11,12^ despite the technical challenges posed by thrombocytopenia. Consequently, post-procedural thrombosis prevention requires particular vigilance in this high-risk subgroup, balancing thromboembolic protection against haemorrhagic complications.^13^ A comprehensive, heart team-based approach is essential to optimize outcomes in such high-risk patients.

## Conclusion

This case confirms the feasibility of TEER in patients with massive left atrial enlargement and severe mixed-aetiology MR, despite high anatomical complexity. Success relied on precise transseptal puncture and a haemodynamically guided multi-clip strategy, achieving significant regurgitation reduction without compromising left ventricular filling. The procedure resulted in marked symptomatic improvement and left atrial reverse remodelling, supporting an integrated haemodynamic approach over a strictly anatomic one.

## Supplementary Material

ytag222_Supplementary_Data

## Data Availability

The data supporting this case report are available within the article and its online Supplementary material.

## Lead author biography

Dr Ni Qingwei, MD, PhD, graduated from Wenzhou Medical University and currently works at the First Affiliated Hospital of Wenzhou Medical University, specializing in transcatheter heart valve interventions.
